# Genome-wide association study identifies common and low-frequency variants at the *AMH* gene locus that strongly predict serum AMH levels in males

**DOI:** 10.1093/hmg/ddv465

**Published:** 2015-11-24

**Authors:** John R.B. Perry, George McMahon, Felix R. Day, Susan M Ring, Scott M. Nelson, Debbie A. Lawlor

**Affiliations:** 1 MRC Epidemiology Unit, University of Cambridge School of Clinical Medicine, Box 285 Institute of Metabolic Science, Cambridge Biomedical Campus, Cambridge CB2 0QQ, UK,; 2 MRC Integrative Epidemiology Unit at the University of Bristol, Bristol BS8 2BN, UK,; 3 School of Social and Community Medicine, University of Bristol, Oakfield House, Oakfield Grove, Bristol BS8 2BN, UK and; 4 School of Medicine, University of Glasgow, GlasgowG31 2ER, UK

## Abstract

Anti-Müllerian hormone (AMH) is an essential messenger of sexual differentiation in the foetus and is an emerging biomarker of postnatal reproductive function in females. Due to a paucity of adequately sized studies, the genetic determinants of circulating AMH levels are poorly characterized. In samples from 2815 adolescents aged 15 from the ALSPAC study, we performed the first genome-wide association study of serum AMH levels across a set of ∼9 m ‘1000 Genomes Reference Panel’ imputed genetic variants. Genetic variants at the *AMH* protein-coding gene showed considerable allelic heterogeneity, with both common variants [rs4807216 (*P*_Male_ = 2 × 10^−49^, Beta: ∼0.9 SDs per allele), rs8112524 (*P*_Male_ = 3 × 10^−8^, Beta: ∼0.25)] and low-frequency variants [rs2385821 (*P*_Male_ = 6 × 10^−31^, Beta: ∼1.2, frequency 3.6%)] independently associated with apparently large effect sizes in males, but not females. For all three SNPs, we highlight mechanistic links to *AMH* gene function and demonstrate highly significant sex interactions (*P*_Het_ 0.0003–6.3 × 10^−12^), culminating in contrasting estimates of trait variance explained (24.5% in males versus 0.8% in females). Using these SNPs as a genetic proxy for AMH levels, we found no evidence in additional datasets to support a biological role for AMH in complex traits and diseases in men.

## Introduction

The gonadal hormone anti-Müllerian hormone (AMH) is an essential messenger of sexual differentiation. In the male foetus, AMH results in involution of the Müllerian ducts, allowing Wolffian ducts to develop into the male reproductive tract under the influence of testosterone. In the female foetus, the absence of AMH allows the Müllerian duct to differentiate into uterine-fallopian internal reproductive structures. Pathological disruption of AMH activity in XY karyotype males leads to the persistent Müllerian duct syndrome, a rare condition in which atypical development of the uterus and sometimes other Müllerian structures occurs (1,2). Rare mutations underlying this autosomal recessive disorder have been mapped to the AMH protein-encoding gene (*AMH*) and its receptor (*AMHR2*); however, ∼15% of cases are idiopathic (3).

In recent years, the development of sensitive assays for AMH has allowed many studies of the role of postnatal AMH levels (which are ∼10-fold lower than in the foetus) as a gonadal marker in both sexes. In pre-pubertal boys, measurement of serum AMH is performed in clinical settings as a reliable indicator of functional testes; its absence supports the diagnosis of primary hypogonadism (4–5). In women, serum AMH has emerged as the most robust circulating biomarker of reproductive lifespan (6). Produced by ovarian granulosa cells, AMH levels are highly correlated with ovarian primordial follicle number and levels gradually decline with age until becoming undetectable ∼5 years before menopause when the follicle stock is depleted (7–10).

In epidemiological studies, the association of serum AMH levels with a broad range of cardio-metabolic traits in women has been assessed, with conflicting results suggesting higher levels are associated with increased disease risk (11,12), reduced risk (13,14) and null associations (15–18). However, in all cases the causal nature of these relationships is unknown, not least because the associations have been cross-sectional, predominantly in small numbers and selected groups with polycystic ovary syndrome, sub-fertility or assessed during pregnancy. Genetic studies have the potential to aid these epidemiological analyses as associations with genetic variants are not subject to confounding or reverse causality (19). Where available, such genetic variants have been analysed as proxy instruments to make valuable inferences regarding the likely causal associations. However, few large studies have measured AMH levels and GWAS data, and there are as yet no common genetic variants identified with robust associations with serum AMH levels that could be used as instrumental variables to assess causal associations.

Here, we report the first genome-wide association study (GWAS) of serum AMH levels in a sample of 1360 males and 1455 females from the ALSPAC birth cohort study. We identified several genetic variants at the *AMH* locus, which explain a substantial proportion of the population variance in AMH levels in males, but not in females. Following on from this, we completed a phenome scan in both ALSPAC males and deCODE (males and females combined) studies to generate hypotheses around potential causal effects of AMH on a range of phenotypes, which found no evidence for a causal role in over 2500 human disease/traits tested.

## Results

### A GWAS for serum AMH levels in adolescents

**Figure 1.**
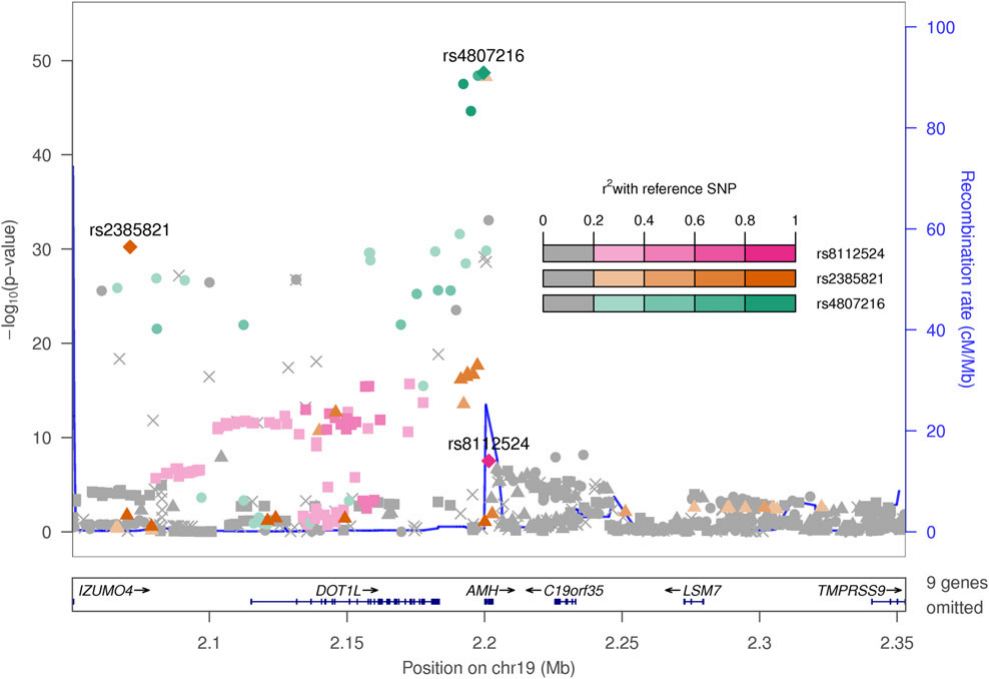
Regional association plot at the *AMH* gene region.

Natural log-transformed serum AMH levels were higher in 1360 males compared with 1455 females from the ALSPAC study (Supplementary Material, Table S1). The GWAS for logged AMH levels in all 2815 adolescents (females and males combined) identified a total of 113 variants associated with serum AMH levels beyond the genome-wide significance threshold, all of which were located in/near the *AMH* protein-coding gene (Table 1 and Fig. 1). The lead SNP was located 430 bp downstream of *AMH* [mean difference in AMH = ∼0.34 standard deviations (SD) per allele, *P* = 1.8 × 10^−18^].

**Table 1.** Genome-wide significant associations observed at the AMH gene locus

	SNP	Distance^a^	Format^b^	Univariate^c^		Joint model^d^		GCTA model^e^		Variance (%)^f^
				Beta^g^	*P*	Beta	*P*	Beta	*P*	
All	rs4807216	430 bp	C/T/0.14	0.25 (0.03)	1.8E–18	0.33 (0.04)	3.E–20	0.34 (0.04)	2.4E–21	5.60
	rs2385821	129 kb	G/A/0.964	0.16 (0.05)	0.002	0.51 (0.06)	5.E–18	0.53 (0.06)	3.9E–18	
	rs8112524	Intronic	G/A/0.39	0.16 (0.02)	6.7E–13	0.08 (0.03)	2.E–03	0.09 (0.03)	0.0004	
Males	rs4807216	430 bp	C/T/0.14	0.46 (0.03)	7.4E–50	0.53 (0.04)	2.E–49	0.56 (0.04)	6.0E–45	24.50
	rs2385821	129 kb	G/A/0.964	0.14 (0.06)	0.02	0.7 (0.06)	6.E–31	0.78 (0.07)	1.9E–28	
	rs8112524	Intronic	G/A/0.39	0.3 (0.02)	2.0E–34	0.15 (0.03)	3.E–08	0.18 (0.03)	1.3E–09	
Females	rs4807216	430 bp	C/T/0.14	0.01 (0.04)	0.67	0.09 (0.05)	7.E–02	0.08 (0.04)	0.06	0.80
	rs2385821	129 kb	G/A/0.964	0.17 (0.06)	0.008	0.25 (0.07)	8.E–04	0.25 (0.07)	0.0009	
	rs8112524	Intronic	G/A/0.39	0.01 (0.03)	0.81	0.002 (0.03)	9.E–01	−0.001 (0.03)	0.97	

^a^Distance from AMH protein-coding gene.

^b^Effect allele/other allele/effect allele frequency.

^c^Unadjusted test statistics from the primary genome-wide association scan.

^d^Effect estimates when all three SNPs are jointly included in the regression model.

^e^Effect estimates when all three SNPs are jointly included in the GCTA approximate conditional analysis framework.

^f^Trait variance explained by all three SNPs.

^g^All effect estimates based on natural log-transformed ng/ml serum AMH.

### Independent signals at the AMH gene locus

Given the large number of variants associated at this locus with a broad range of allele frequencies (MAF 2–40%), we tested for the presence of multiple statistically independent signals at the *AMH* locus. An analysis conditioning on the effect of the lead SNP (rs4807216) revealed an additional low-frequency SNP (rs2385821) at genome-wide significance (Table 1). This second SNP was in moderate LD (*r*^2^ = 0.22) with the lead index SNP, but its addition to a joint model increased the variance explained in serum AMH from 2.7% by rs4807216 alone to 5.2% by both SNPs.

### AMH-associated variants exhibit sex-specific effects

We next aimed to identify variants with sex-specific effects on AMH by performing two stratified analyses in the subsets of 1360 males and 1455 females. No variants reached genome-wide significance in females; however, a strong signal, represented by the same lead SNP as in the combined analysis, was observed in males (Table 1; rs4807216, *P* = 7 × 10^−50^). A test for heterogeneity revealed a striking sex interaction for this lead SNP [*P*_Het_ = 9.3 × 10^−22^], which was not associated with AMH levels in females (*P* = 0.67).

We repeated conditional analyses in the sex-specific strata and found three independent signals at the *AMH* gene locus in males (Table 1), only one of which showed any evidence of even potential association with AMH levels in females (rs2385821, *P* = 8 × 10^−4^). In a joint model, all three SNPs demonstrated significant heterogeneity between sexes (rs4807216 *P*_Het_ = 6.3 × 10^−12^, rs2385821 *P*_Het_ = 1 × 10^−6^ and rs8112524 *P*_Het_ = 0.0003), with widely contrasting estimates of the phenotypic variance explained in a model using all three SNPs: 24.5% in males versus 0.8% in females. To examine whether sex differences in AMH levels and/or variability influenced the gene × sex interactions, we examined a sub-sample of 713 boys and girls matched for an identical AMH mean and SD. These demonstrated a very similar pattern of sex-specific associations (results available from authors on request).

We sought to confirm the identification of these statistically independent signals using an ‘approximate conditional analysis’ methodology implemented in GCTA (20), run only on summary level statistics. Highly concordant effect estimates were observed in a GCTA joint model (Table 1), further highlighting the utility of this methodology.

### Functional assessment of AMH-associated SNPs

To identify the potential mechanism(s) linking SNP association to *AMH* function, we assessed publicly available gene expression data from the GTex consortium (www.gtexportal.org), in addition to searching for correlated non-synonymous variants. All three SNPs were associated with *AMH* mRNA transcript levels in multiple tissue types (Supplementary Material, Table S3, *P*_min_ 6 × 10^−5^–1 × 10^−15^). The lead SNP for AMH protein levels (rs4807216) was also the same lead SNP for the strongest genome-wide signal for AMH mRNA levels (skeletal muscle, *P* = 1 × 10^−15^); however, for rs4807216 (and also rs8112524) the mRNA increasing allele was associated with decreased protein levels, despite directionally concordant effects on mRNA across all significant tissues. Two of the three SNPs were correlated (*r*^2^ > 0.5) with missense variants in *AMH* (rs4807216 *r*^2^ = 0.54 with rs10407022; rs2385821 *r*^2^ = 0.55 with rs10417628).

### Mendelian randomization phenome-wide scan of AMH-associated variants

We performed a ‘Mendelian Randomisation’ analysis to test the likely causal relevance of circulating AMH levels on a broad range of human disease and health-related traits. Using our newly identified SNPs as a genetic proxy for AMH, we found no evidence for a causal relationship between AMH and complex selected available traits in the ALSPAC males (trait *N* = 30, *P* > 0.002; Supplementary Material, Table S2). Nor did we find any evidence of association in deCODE (males and females combined; trait *N* = 2528, *P* > 2 × 10^−5^; Supplementary Material, Figs. S1 and S2).

## Discussion

Our study represents the first large-scale effort to elucidate the genetic architecture of circulating AMH levels. As with other sex hormones, progress in identifying genetic determinants has been relatively slow compared with other complex traits, primarily due to a paucity of data. Other studied sex hormones (and their binding proteins) include testosterone (21,22), dehydroepiandrosterone sulphate (23) and sex hormone binding globulin (SHBG) (24). In keeping with several of these studies, we demonstrate a strong *cis* effect of variants at the protein-coding gene locus, including evidence for multiple independent regulatory elements. We also demonstrate sex-differentiated effects; however, we note that the dichotomy observed between sexes in our study is stronger than any previously reported for a genetic effect on a sex hormone. We hypothesize that the relative lack of polygenicity in male AMH reflects its non-essential role in adult health, with no strong selection acting on these alleles. A total of three statistically independent signals were identified through formal conditional analysis, with highly concordant results obtained through approximate conditional analysis. We found that two of the three identified signals were in high LD with *AMH* missense alleles, one of which has been experimentally demonstrated to influence bioactivity of the *AMH* protein (25). Each of these was also associated with *AMH* gene expression in multiple tested tissues. Paradoxically the expression raising allele decreased protein levels for two of these signals; however, there are a number of likely explanations, such as effects on RNA splicing leading to differential isoform expression and numerous post-transcriptional and translational events affecting processes such as protein secretion into the circulation. In aggregate these data convincingly link our genetic variation to *AMH* gene function; however, further fine-mapping studies and experimental evidence are required to elucidate the molecular mechanism(s).

Aside from insight into the genetic regulation of AMH, the identification of associated genetic variants has utility to inform broader biomedical questions. The principles of ‘Mendelian Randomization’ have been widely utilized to model genetic variants as non-confounded proxies for traits of interest (26–31). Notable examples in the context of reproductive biology include causal effects of SHBG on T2D (32,33) and BMI on age at menarche (34,35). Given the relatively large effect of AMH-related SNPs on male AMH levels (explaining 25% of its variation), we can conclude that it is unlikely that circulating AMH levels are a strong influencing factor for common traits and disease outcomes in men.

Given the relatively weak genetic effect in females, we were unable to assess what further role circulating levels may have on the tested health outcomes in females. While the phenome scan in deCODE is undertaken in females and males combined (due to our access to these combined summary data only), the results should be interpreted as reflecting a Mendelian randomization study in males only since the genetic instrument explains very little variation in AMH in females. Currently AMH testing is used in a number of diagnostic settings, including for ovarian reserve measurement where it remains a leading predictive biomarker test (36,37). Biomarkers that are good predictors of an outcome are not necessarily causally related. Future studies identifying and assessing the effect of genetic variation on AMH levels may look to integrate such genetic data into these diagnostic tests to improve predictive power. This approach has been demonstrated for PSA levels, where genetic correction for the biomarker improved prostate cancer diagnosis (38). The relatively small effect of our identified variants on female AMH levels likely excludes this as a possibility until a time when additional genetic determinants have been identified.

We note a number of limitations of this study. First, due to a paucity of available data, we were unable to replicate our identified genetic signals. However, the highly relevant nature of the gene locus and our closely supporting functional evidence provide confidence that these are real signals for AMH levels. Exaggeration of the true effect sizes due to ‘winners curse’ may be present, and it will be important for future studies to better estimate the true effect in both sexes. However, even if the true effect estimate was half the size observed here, the genetic variants would still explain >10% of AMH variability in males. Second, although two separate statistical approaches revealed evidence for at least three independent effects, it remains possible that a smaller number of untyped causal variants could explain this apparent allelic heterogeneity (39). This can ultimately only be resolved through fine-mapping approaches; however, the relatively high degree of coverage provided by the 1000 genomes reference panel reduces this possibility. Third, AMH was determined when participants were largely postpubertal, and although in males this is likely to reflect adult levels, in females AMH continues to increase until approximately age 25 years (40). Replication of our findings in older cohorts is warranted to exclude any age-specific genotype effects. Finally, the hypothesis-free phenome scan in deCODE was restricted to a sex-combined analysis using only the lead AMH SNP. This limitation, along with the conservative Bonferroni correction threshold applied to the tested diseases/traits, reduces our power to identify possible subtle effects of AMH on health-related outcomes and, as noted earlier, we would interpret these null results as being generalizable to males only.

In summary, these genome-wide analyses have identified the first genetic variants robustly associated with serum AMH levels. Given their relatively large effects, we were able to use these variants as genetic instruments to infer that variability in AMH levels is unlikely to substantially contribute to complex traits and disease susceptibility in males.

## Methods

The Avon Longitudinal Study of Parents and Children (ALSPAC) is a longitudinal, population-based birth cohort that recruited 14 541 pregnant women resident in Avon, UK, with expected dates of delivery 1 April 1991 to 31 December 1992 (http://www.alspac.bris.ac.uk.) (41,42). Since age 7, surviving offspring participants have been invited to regular follow-up clinics. A total of 5515 adolescents attended the 14–16 (mean age 15 years) clinic, of which 5376 were singletons; only singletons are considered here. Detailed summary statistics of the AMH phenotyped study population have been described elsewhere (18,43).

Ethical approval was obtained from the ALSPAC Law and Ethics Committee (IRB 00003312) and the Local Research Ethics Committee. The study website contains details of all the data that is available through a fully searchable data dictionary (http://www.bristol.ac.uk/alspac/).

### Genotyping

Within ALSPAC, the index child participants, studied here, were genotyped using the Illumina HumanHap550 quad genome-wide SNP genotyping platform by Sample Logistics and Genotyping Facilities at the Wellcome Trust Sanger Institute and LabCorp (Laboratory Corportation of America) using support from 23andMe. Participants were excluded on the basis of having incorrect sex assignments; minimal or excessive heterozygosity (<0.32 and >0.345 for the Sanger data and <0.31 and >0.33 for the LabCorp data); disproportionate levels of individual SNP missingness (>3%); evidence of cryptic relatedness (>10% IBD) and being of non-European ancestry. EIGENSTRAT analysis revealed no additional obvious population stratification, and genome-wide analyses with other phenotypes indicate little genomic inflation of association estimates in this population. The resulting dataset consisted of 8365 individuals. SNPs with a minor allele frequency of <1% and call rate of <95% were removed. Furthermore, only SNPs that passed Exact tests of Hardy–Weinberg equilibrium (*P* > 5 × 10^−7^) were considered for analysis. We combined 477 482 SNPs genotypes of these subjects with genotype data of a sample of 9048 mothers to allow the use of a large number of duos by the phasing algorithm to increase quality of phased haplotypes and downstream imputation. This resulted in the removal SNPs genotypes with a poor concordance across datasets, which could be completely captured by increasing the missingness filter 5–1% (an additional 11 396 SNPs). We also removed a further 321 subjects due to potential id mismatches when assessed using duo data. We estimated haplotypes using ShapeIT (v2.r644), which utilizes relatedness during phasing. We imputed with Impute2 v2.2.2 using the 1000 genomes phase 1 version 3 reference data panel. This gave 8237 eligible adolescents with available genotype data after exclusion of related subjects using cryptic relatedness measures described previously.

### AMH measurement

Participants were asked to fast overnight, for those attending in the morning, or for a minimum of 6 h, for those attending in the afternoon. Following venepuncture, blood samples were immediately spun, plasma separated, and frozen at −80°C. AMH was assayed in heparin plasma, after a median 10 months storage with no freeze-thaw cycles, using the commercial AMH Generation II ELISA kit (Beckman Coulter UK Ltd, High Wycombe, UK) (43,44). Inter and intra-assay CVs were both <5%.

### Association testing

Serum AMH levels were natural log-transformed prior to analysis with outliers ±4 SDs removed, approximating a normal distribution (Supplementary Material, Table S1). Association testing was performed using a linear regression model implemented in SNPTest v2.5. The analysis was run three times—in males only, in females only and in all individuals with sex as a covariate. Significance was declared at the genome-wide threshold of *P* < 5 × 10^−8^. Independent genetic signals were identified using two approaches—approximate conditional analysis implemented in GCTA using UK10K genotypes as a reference panel; and formal conditional analyses were performed by re-running the GWAS with the inclusion of the tested SNP(s) as covariates.

### Mendelian randomization

Using our newly identified genetic variants as a proxy instrument for circulating AMH levels, we performed Mendelian Randomization analyses using two complementary approaches. First, male specific analyses were performed across 30 biologically plausible outcomes in the ALSPAC study (Supplementary Material, Table S1). The number of copies of the reference allele of each of the three AMH-related SNPs identified after association testing were weighted by their adjusted effect size and direction of effect in PLINK (v1.07). SNP rs4807216 reference allele C was weighted by 0.533, SNP rs2385821 reference allele G was weighted by 0.699 and SNP rs8112524 reference allele G was weighted by 0.145. Each individual was thereby scored as a weighted sum of the number of reference alleles for these three SNPs, and the resulting values were used as an independent variable in a linear regression of health-related outcomes.

Second, we utilized a hypothesis-free approach by testing the association of the most strongly associated AMH SNP with 2528 traits or diseases in a sex-combined cohort from the deCODE study (45), with a maximum trait sample size of 104 220. A conservative significance threshold was declared at a Bonferroni level of *P* < 0.05/N traits tested.

## Author contributions

D.A.L. and J.R.B.P. designed the study, D.A.L., S.M.R. and S.M.N. obtained funds for and were involved in phenotypic data collection, G.M. undertook analyses in ALSPAC, J.R.B.P. wrote the first draft of the paper with input from F.R.D. and D.A.L., all the authors contributed critical revisions of the paper and approved the final version.

J.R.B.P. and D.A.L. will serve as guarantors for the contents of this paper.

## Supplementary Material

Supplementary Material is available at *HMG* online.

## Funding

The UK Medical Research Council and the Wellcome Trust (Grant ref: 102215/2/13/2) and the University of Bristol provide core support for ALSPAC. The work in this paper is funded by a grant from the US National Institute of Health (R01 DK077659). D.A.L., S.M.R. and G.M. work in a Unit that receives funds from the UK Medical Research Council (MC_UU_12013/5) and the University of Bristol. D.A.L. is a UK NIHR Senior Investigator (NF-SI-0611-10196).

This publication is the work of the authors and not necessarily any of the funding bodies who supported the work. Funding to pay the Open Access publication charges for this article was provided by the Medical Research Council and the Wellcome Trust.

## Supplementary Material

Supplementary Data
