# Supplementary material for: Genome-wide association study identifies common and low-frequency variants at the AMH gene locus that strongly predict serum AMH levels in males
Source: Hum Mol Genet. 2015 Nov 24;25(2):382–8. doi: 10.1093/hmg/ddv465 (PMC4706112; doi:10.1093/hmg/ddv465)
Supplement: Supplementary Data [file supp_25_2_382__index.html]

Genome-wide association study identifies common and low-frequency variants at the AMH gene locus that strongly predict serum AMH levels in males — Genome-wide association study identifies common and low-frequency variants at the AMH gene locus that strongly predict serum AMH levels in males — Supplementary Data 

# Genome-wide association study identifies common and low-frequency variants at the *AMH* gene locus that strongly predict serum AMH levels in males

## Supplementary Data

Supplementary Data

- Supplementary Figures - docx file
- Supplementary Tables - xlsx file
