# Supplementary material for: Genome-wide association study identifies common and low-frequency variants at the AMH gene locus that strongly predict serum AMH levels in males
Source: Hum Mol Genet. 2015 Nov 24;25(2):382–8. doi: 10.1093/hmg/ddv465 (PMC4706112; doi:10.1093/hmg/ddv465)
Supplement: Supplementary Data [file supp_ddv465_ddv465supp_figs.docx]

**Supplementary Figure 1 | Quantile-quantile plot of the SNP rs4807216 against 894 quantitative traits measured in the deCODE study**


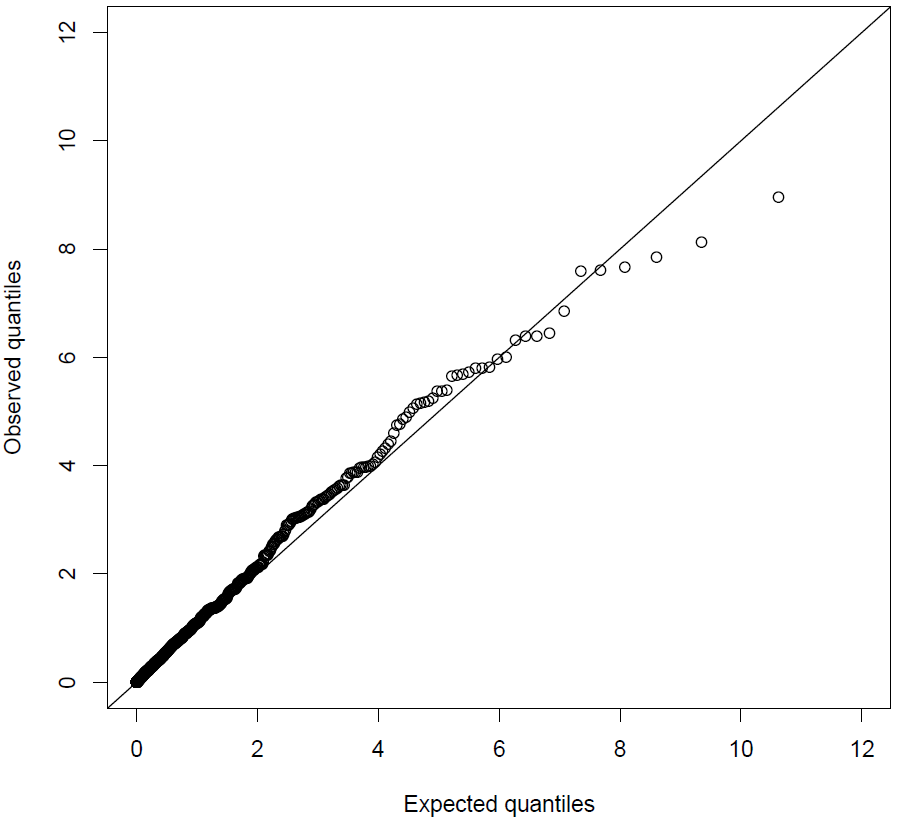


**Supplementary Figure 2 | Quantile-quantile plot of the SNP rs4807216 against 1634 dichotomous outcomes measured in the deCODE study**

**
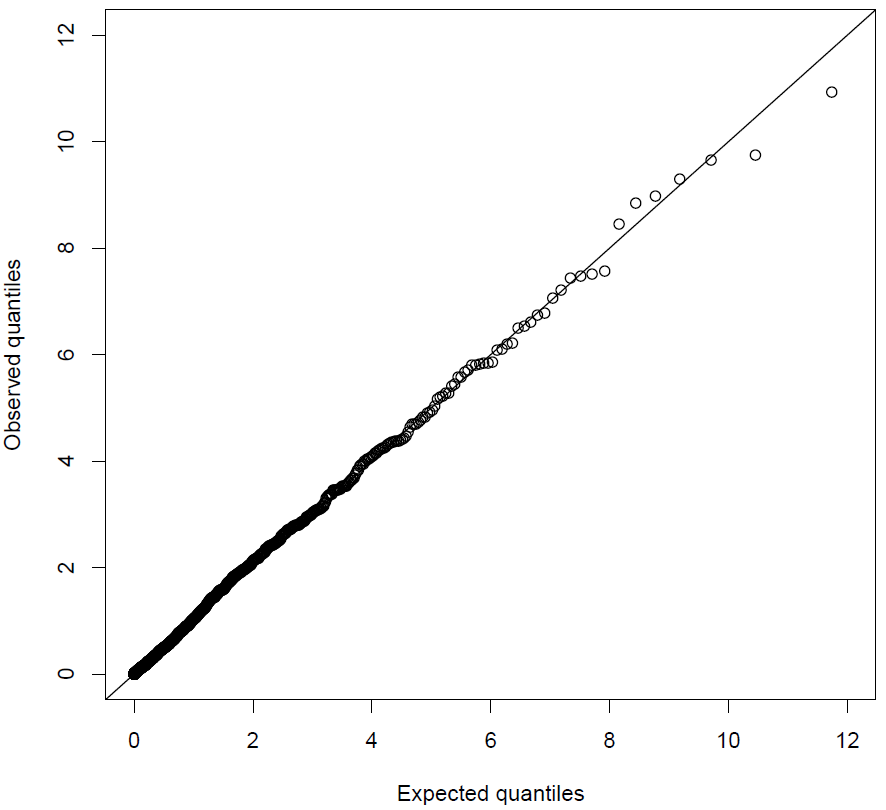
**
